# Health Impact Assessment in New South Wales & Health in All Policies in South Australia: differences, similarities and connections

**DOI:** 10.1186/1471-2458-14-699

**Published:** 2014-07-09

**Authors:** Toni Delany, Patrick Harris, Carmel Williams, Elizabeth Harris, Fran Baum, Angela Lawless, Deborah Wildgoose, Fiona Haigh, Colin MacDougall, Danny Broderick, Ilona Kickbusch

**Affiliations:** 1 Southgate Institute for Health Society and Equity, Flinders University, Adelaide, Australia; 2 Centre for Health, Equity, Training, Research and Evaluation, University of New South Wales, Sydney, Australia; 3 Department of Health and Ageing, SA Health, Adelaide, Australia; 4 Discipline of Public Health, Flinders University, Adelaide, Australia; 5 Graduate Institute of International and Development Studies, Geneva, Switzerland

**Keywords:** Health in all policies, Health impact assessment, Healthy public policy

## Abstract

**Background:**

Policy decisions made within all sectors have the potential to influence population health and equity. Recognition of this provides impetus for the health sector to engage with other sectors to facilitate the development of policies that recognise, and aim to improve, population outcomes. This paper compares the approaches implemented to facilitate such engagement in two Australian jurisdictions. These are Health Impact Assessment (HIA) in New South Wales (NSW) and Health in All Policies (HiAP) in South Australia (SA).

**Methods:**

The comparisons presented in this paper emerged through collaborative activities between stakeholders in both jurisdictions, including critical reflection on HIA and HiAP practice, joint participation in a workshop, and the preparation of a discussion paper written to inform a conference plenary session. The plenary provided an opportunity for the incorporation of additional insights from policy practitioners and academics.

**Results:**

Comparison of the approaches indicates that their overall intent is similar. Differences exist, however, in the underpinning principles, technical processes and tactical strategies applied. These differences appear to stem mainly from the organisational positioning of the work in each state and the extent to which each approach is linked to government systems.

**Conclusions:**

The alignment of the HiAP approach with the systems of the SA Government increases the likelihood of influence within the policy cycle. However, the political priorities and sensitivities of the SA Government limit the scope of HiAP work. The implementation of the HIA approach from outside government in NSW means greater freedom to collaborate with a range of partners and to assess policy issues in any area, regardless of government priorities. However, the comparative distance of HIA from NSW Government systems may reduce the potential for impact on government policy. The diversity in the technical and tactical strategies that are applied within each approach provides insight into how the approaches have been tailored to suit the particular contexts in which they have been implemented.

## Background

Public health has a long history of recognising the importance of factors located outside of the control of the health system [1]. The last few years have seen intensified interest in strategies which influence policies across all sectors in order to improve population health and equity [2-7]. Such interventions require the health sector to work intersectorally, particularly within government, to advocate for health, enable health promoting activities and mediate between differing interests across sectors [2,8-12]. While applying an intersectoral approach is not easy, this work can be facilitated through the application of processes that encourage the formation of relationships to collaboratively examine policies and plans [13,14]. This paper examines approaches to intersectoral action for health, which have been applied in two Australian jurisdictions. These are Health Impact Assessment (HIA) in New South Wales, applied since 2003 [15] and Health in All Policies (HiAP) in South Australia, applied since 2007 [7,16,17].

## Purpose and method

We argue that many of the principles, techniques and strategies that are applied as part of the HIA and HiAP are similar. However, the analysis also reveals differences, particularly regarding the positions of those who implement the work in each state, the different institutional conditions in the jurisdictions, and the different tactical and technical approaches that these necessitate. This examination makes an important contribution to the current literature where HIA and HiAP approaches have tended to either be conflated as if they are the same or discussed separately, thereby reducing opportunity for analysis of their differences, similarities and points of connection [18]. Furthermore, there has been increasing global interest in both HiAP and HIA, but a lack of clarity about how these are applied in specific contexts [19,20]. Consideration of this is important given the considerable diversity in how HIA and HiAP are practiced internationally [19,21,22].

Analysis for this paper was strengthened by explicitly focussing, in line with established policy analysis theory [23], on how HIA and HiAP fit within the policy and planning cycle and within the subsystems that create policy in each jurisdiction. The ideas presented in this paper have emerged through collaborative activities between the stakeholders in both jurisdictions. The first of these involved discussion and writing between researchers from SA (TD, FB) and members of the SA Health in All Policies team following attendance at a workshop on the NSW HIA approach in 2012. This collaborative work resulted in the generation of tables that compared and contrasted the HiAP and HIA approaches. In 2013, academics (TD, FB, AL) and HiAP staff (CW, DW) from South Australia and HIA practitioners and academics from NSW (PH, EH, FH) held a workshop to compare the two approaches. The results from this work were later progressed through a discussion paper written to inform a plenary session at the *International Union of Health Promotion and Education World Conference on Health Promotion* in Thailand [24]. During the development of this discussion paper, the authors drew upon their experiences of working with the HiAP/HIA approaches and the tacit knowledge derived from this. The authors also drew on more formal sources, such as reports about particular HIA and HiAP projects. The large international audience of practitioners and academics at the plenary session provided feedback on the ideas presented and the authors drew on these, as well as relevant policy theory, to strengthen the analytic rigour [25] of the analysis. The paper begins with a description of each approach and the contexts in which they operate. The processes applied in both approaches are then compared and contrasted, with consideration of the many points of connection provided throughout.

## Results and discussion

### Broader context

#### Health Impact Assessment (HIA): New South Wales

HIA has been undertaken in NSW since 2003. In NSW, HIA has predominantly been developed and applied by the Centre for Health Equity Training, Research and Evaluation (CHETRE) at the University of NSW who use HIA in partnership with both government and non-government collaborators [26].

HIA offers a structured, step wise process for the assessment and prediction of the potential health impacts of policies, plans, programs and projects [18]. HIA, as it has developed in NSW, has tended to occur outside a legislative framework and without a central mandate. It operates mainly as a decision-support process whereby agencies or organisations, often from within government, undertake or commission a HIA [21,27]. To date, the majority of HIAs in NSW have focussed on plans, for example draft health service plans, or plans related to urban development [28]. The majority of HIAs in NSW have followed a learning by doing approach, where an agency or organisation has been supported by CHETRE to do the HIA while learning about the process as this unfolds [29]. Additionally, CHETRE has led some HIAs, often with an explicit equity focus [30].

#### Health in All Policies (HiAP): South Australia (SA)

Health in All Policies (HiAP) was introduced in SA in 2007. The approach provides a foundation for public servants from the Health sector to work with those in other sectors of the SA Government to consider the potential health and wellbeing implications of policies as they are conceptualised, developed and implemented [6,31]. HiAP work is linked strongly to *South Australia’s Strategic Plan* (SASP) [32], which calls for ‘joined-up’ government approaches that work across departments to achieve specified targets and objectives. SA’s HiAP model is also applied as a learning by doing approach through reflection on, and formal evaluation of, HiAP practice as it proceeds [4]. HiAP in SA has a mandate from the Premier and Cabinet, which provides central governance, commitment and accountability [31]. Since its implementation, the HiAP approach has adapted to South Australia’s changing political context in order to ensure that it remains relevant and useful [33]. An example of this is the effort made to link HiAP projects to the Seven Strategic Priorities of Government [34] during 2012 and 2013. Until late 2013, HiAP was operationalised by a core group of staff in a dedicated HiAP unit. Part of their work involved the development and application of a practical methodology that supports the HiAP approach, called the Health Lens Analysis (HLA) process [6,31]. Application of the HLA process is continuing as part of the work of a broader Public Health Partnerships Branch in order to institutionalise the approach and align it with the *South Australian Public Health Act*[35]. HiAP in SA has also attracted bi-partisan support, which may contribute to its strength [36]. Essential information about the broader context that HiAP operates in, and how this compares to that of the NSW HIA approach, is provided in Table 1.

**Table 1:** The broader contexts of HIA in NSW and HiAP in SA

	**New South Wales: Health Impact Assessment**	**South Australia: Health in All Policies**
Government system	The NSW Government serves 7,000,000 people, most of whom (approximately 67%) live in the urban centre of Sydney.	The SA Government serves 1,500,000 people, most of whom (approximately 77%) live in the urban centre of Adelaide.
	There have been several changes in Government leadership since 2001, with 4 different Labor Premiers between 2001 and 2011 and the Liberal Party forming Government in 2011.	Relatively stable Labor Government since 2002, with only one change in Premiership. In March 2014 Labor was re-elected with support from an Independent to form a minority government in SA.
Government support	No current central mandate for HIA – historically support from within the health system.	Explicit support, galvanised early on by the ‘Thinkers in Residence’ program. HiAP program tied to policy making processes, formal State strategic plan, governance structures and machinery of government. Bipartisan support also evident [36].

### Organisational positioning of the approaches

One of the key differences that exist between HIA in NSW and HiAP in SA relates to the organisational position of those involved. HIA is primarily implemented through collaborations between a university based institution *outside* government and practitioners inside state government and local governments as well as with community groups and NGOs. HiAP is implemented by public servants who work *within* the SA Department of Health and Ageing who collaborate with public servants from other state government, and, increasingly, local government, departments. This influences the principles, techniques and strategies that are applied as part of each approach.

#### Principles underpinning HiA and HiAP

HIA’s principles stem from, and are guided by, the broad societal values of democracy, sustainability and equity [37,38]. The NSW approach draws on these principles and the work is focussed particularly on achieving a more equitable society where health and wellbeing outcomes are optimised. The initial driver for HIA’s introduction in NSW was to build the capacity of the NSW health system to address equity internally and collaboratively with other sectors [15]. Since its introduction, HIA in NSW has not been linked to any overarching Government principles or strategies but has rather taken the form of a structured method to assess and predict the impact of proposals – within health and other sectors. As such, the HIA approach is now driven by a range of stakeholders both inside and outside of Government and is applied to topics of interest to these various stakeholders.

The principles underpinning HiAP in SA have stemmed from, and are informed by, the key drivers of the South Australian Government. As highlighted earlier, one such driver is the South Australian Strategic Plan [32], which provides recognition of the need for all sectors to work together to advance social development. This approach is summarised, and linked specifically to health outcomes, in the *Adelaide Statement on Health in All Policies*[39], which was written for an international audience*.* According to the Adelaide Statement, achieving these outcomes requires a new form of governance where there is joined-up leadership within governments, across all sectors and between all levels of government [40]. The Adelaide Statement highlights the changing role of the health sector and indicates that it needs to become a contributor to, and facilitator in, resolving complex problems across government rather than acting only as the leader [40].

#### Collaborative relationships

Both the HIA and HiAP approaches are underpinned by a belief in the value of collaboration to achieve sustainable change in population health outcomes. There is some tactical difference, however, in the emphasis placed on building collaborative relationships. The primary mechanism of the HiAP approach in South Australia is the development of collaborative, internal relationships within Government. These relationships are intended to facilitate the policy process, and through this, to ensure that health concerns are identified and acted upon in policy. Within the HIA approach, relationship building has been viewed as a desirable outcome and facilitator of the approach but not as the explicit goal [28]. This highlights a key difference between the focus of the approaches, which has implications for how the work is undertaken. This is particularly because the focus on relationship building involves HiAP staff spending time building connections and maintaining these over time, whereas this is less of a focus of the work involved in conducting a HIA. Furthermore, because the HiAP approach is focussed strongly on relationship building and is also bounded by the political drivers, political sensitivities and priorities of Government this has the potential to limit the work in a way that the broader approach of HIA may not. This is particularly evident in regard to the way that increasing equity is consistently articulated as an explicit goal of the HIA approach while a focus on equity often remains implicit within the HiAP approach, depending on whether equity is viewed as an acceptable and useful aim within a particular collaboration.

### Points of application in the policy and planning cycle

**Figure 1:**
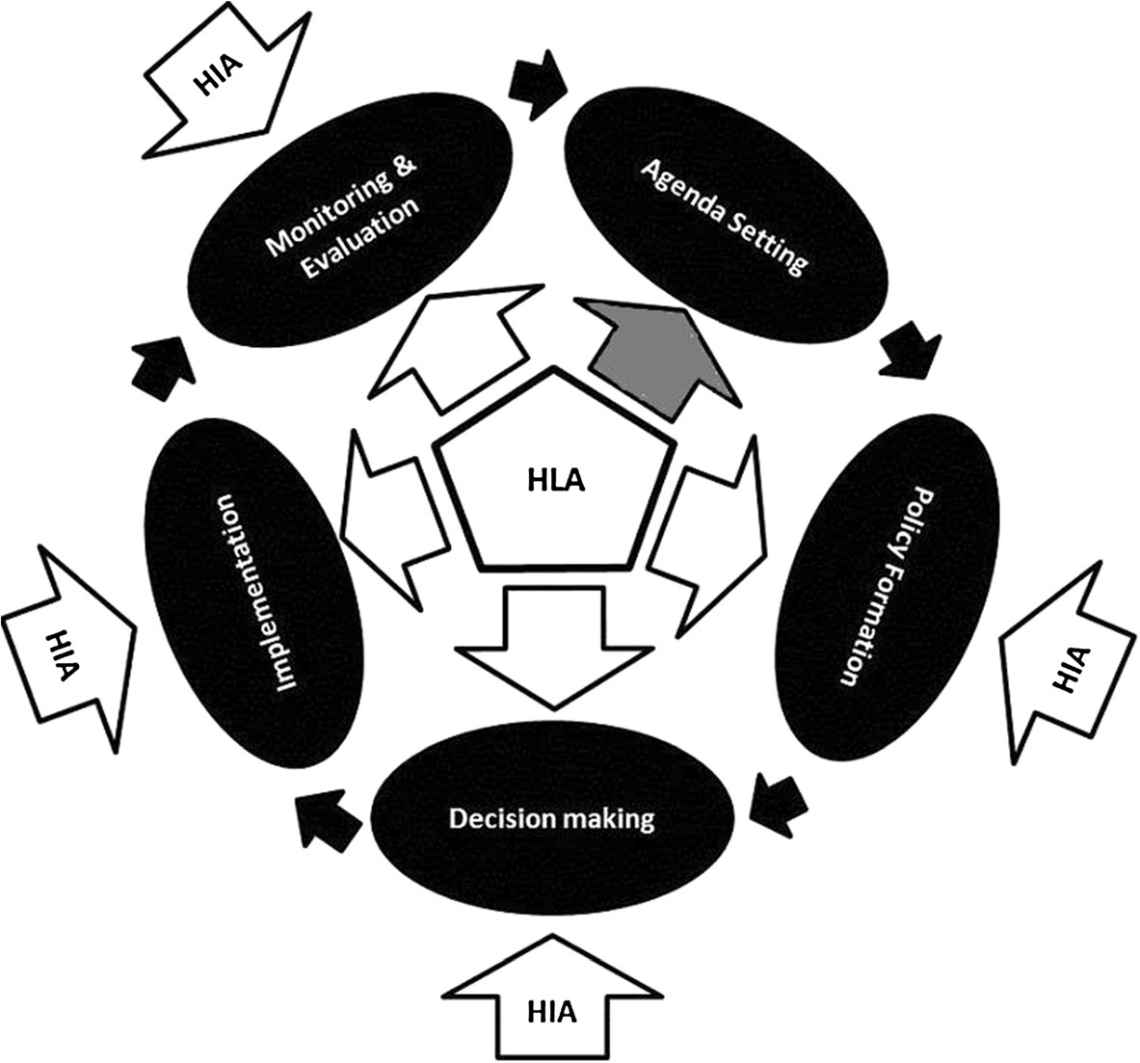
Entry points for HIA and HLA in the policy cycle.

There are differences in the points within the policy cycle at which the two approaches are applied. HIA is typically introduced within the policy and planning cycle [41]*after* a draft proposal has been developed but *before* that proposal is implemented [42]. In practice, there is often a push to conduct HIAs before a draft proposal is fully developed and to adapt HIA flexibly to provide input early and across the life of proposals [20,43]. However, HIA in NSW is usually applied once collaborators have *some* understanding of the issue that a policy or plan will address. For example, a HIA undertaken early in the life of a large scale development on the outskirts of Sydney focussed on assessing a draft plan to develop 12,000 homes. The HIA considered six areas of impact scoped to be of direct relevance to the development (public transport, active transport, social connectivity, physical activity, injury and food access). 24 recommendations were developed and a monitoring process set up to support the implementation of these recommendations. It is for this reason that Figure 1 shows the earliest entry point of a HIA to be at the policy formulation stage of the policy process and not in agenda setting stage.

HiAP almost always engages early in the policy process through application of the Health Lens Analysis (HLA) [31]. Unlike HIA in NSW the HLA can contribute to the *agenda-setting* phase of the policy process [42]. This is facilitated by those who implement the HiAP approach in SA working from *inside* the Government system and having their work determined by the central government agency, Department of the Premier and Cabinet. In the South Australian context HiAP can be understood as a broad, strategic approach with the Health Lens Analysis providing *practical* processes for undertaking intersectoral policy-making.

Another difference concerns intent. HIA aims to influence a specific proposal to consider health and health equity. HIA does this by making causal links between health and the substantive drivers – economic, housing, education, health services and so on - of the sector developing the proposal and which form the basis of the proposed actions in the proposal [20]. For example, HIAs on land use plans specifically link proposed activities like the positioning of local facilities or streetscapes with health outcomes, and HIAs on health service plans draw out the equity implications of developing particular health strategies or service models. These causal links are then used to make recommendations about how proposals can be re-drafted, or further action can be taken, to better consider health and health equity, based on the pathways developed and evidence presented in the HIA.

The primary intent of the HLA process is to highlight the connections and interactions between health and the core business of other sectors. In this way health concerns are positioned as substantive issues which can contribute to the achievement of specific sectoral targets. Thus gains for both health and non-health sectors are identified. Application of the HLA early in the policy process allows health considerations to inform policy at the conceptual stage and may lead to a shift in the policy frame. The evidence developed in the HLAs is then used to inform policy development. [6,31]. There are several examples of where this has occurred within the South Australian context, such as when HiAP staff were provided with a broad brief to work across Government agencies on improving the experience of people who had recently migrated to the state of South Australia to live. Collaborative discussions between HiAP staff and partners from Multicultural SA, the Department for Trade and Economic Development as well as the Attorney General’s Department resulted in multiple project foci being discussed. The project was eventually focussed on identifying how to improve migration settlement outcomes for migrants who are located in rural and regional areas of SA through collecting evidence about how health and wellbeing may be influenced through the experience of settling in these areas. The project produced a report with recommendations for action within each of the partner agencies. The recommendations were intended to shape policies and programs in ways that would improve the health outcomes and settlement experience of migrants. These recommendations were approved by the chief executives of the three partner agencies involved. A process evaluation of the project has also been completed.

Another example stems from the broad brief provided to HiAP staff to work on improving children’s literacy. Discussions between HiAP staff and the Education Department resulted in parental engagement being identified as an area where the Education Department and Health could envisage benefit from additional collaborative work being undertaken. Therefore, the project was focussed on an exploration of how parental engagement with children’s literacy could be encouraged in low socioeconomic areas to improve literacy for children in the early years of schooling, and, through this, ultimately improve their health. The project informed the *South Australian Numeracy and Literacy Strategy 2013*, which, as a result, includes a focus on parental engagement. Parental engagement strategies identified during the project have also been trialled in four schools. Process evaluation of the project is currently being undertaken.

The information in Table 2 summarises, and elaborates on, the differences between the ways that HIA and HiAP are applied.

**Table 2:** Application of HIA and HiAP

	**New South Wales: Health Impact Assessment**	**South Australia: Health in All Policies**
Focus	Works to assess a draft ‘proposal’ (even if this is an idea or an option) to predict its impacts. Is time bound in and around policy formulation and decision making.	Works across the policy making cycle - most often at the beginning of the policy cycle. Is not usually time bound and can be long term. Begins with the identification of a policy area where HiAP can collaborate with other sectors.
Aims of application	Change a proposal by making the links to health by identifying causal pathways between proposed activities, the determinants of health, and health and equity outcomes, and making recommendations for changes in re-drafting the proposal or taking additional action.	Focus on achieving Government core business targets (both those of Health and other departments). Involves identifying causal pathways between health and the determinants under the influence of partnering sectors in a two way dynamic to inform policy development.

### Areas in which HIAs and HiAP HLAs have been undertaken

There is diversity in the areas in which HIA and HiAP have been applied in each state. There has been a particularly broad scope of application in NSW, with HIAs being undertaken by CHETRE in partnership with local health services, local governments and in Government regional offices. Many topics have been assessed through a large number of HIA projects since 2003, with the majority focussing on land use plans and health services development. These include projects funded directly by NSW Health between 2003 and 2009 as part of the *NSW Health Impact Assessment Project* (refer to 44 for a full list of these projects and resulting reports) [44] as well as other HIAs undertaken outside of this project which have been funded by the NSW Government, health services and NGOs and have addressed areas including, but not limited to, the following.

● Equity focused health impact assessments on healthy eating, digital technologies, maternal and child health, chronic condition management, dental health, sexual health, health service development and redevelopment and Medicare Locals.

● HIAs on Indigenous health, various regional and local land use plans and developments, social sustainability, housing and water management.

HiAP’s HLA approach has also been applied to many different policy areas since 2007. These include, but are not limited to, transport, water management, migration, sustainable developments, digital technologies, Indigenous wellbeing, education and training and healthy ageing (refer to 45 for full lists of current and completed HLA projects) [45]. In addition, a HiAP approach has been applied to build capacity in Government agencies to inform work within the government priority areas of *Every Chance for Every Child* and *Safe Communities, Healthy Neighborhoods*. Given the within Government location of HiAP most of the work, to date, has been undertaken within the State Government.

Project topics and partnerships represent another area of difference. Greater freedom is afforded to those undertaking HIAs in NSW to select the topic and recruit collaborating partners. As such, partnerships can, and have, been formed collaboratively between a diverse range of partners, including NGOs, health services and communities to undertake HIAs for a range of purposes, including advocacy and community empowerment [21]. In contrast, the HiAP approach in SA limits formal partners to other government agencies and some policy topics are considered ‘out of bounds’. Community and academic involvement is co-opted for specific purposes – usually in the evidence-gathering phase of the HLA. Community input can be seen as politically risky. Although this limits the scope of activity, the within Government positioning of the HiAP approach also provides opportunities. Specifically, there is potential for HiAP to influence Government decision making directly because the work is focussed on areas of core business for specific government departments. Those undertaking the work are able to use Government systems, such as internal briefings and sign off processes, to highlight findings to senior decision makers directly. The central Government agency mandate that supports HiAP also provides HiAP staff with credibility, which facilitates their entry into other departments. Furthermore, the central mandate, and accompanying governance structure, provides opportunities for staff from the Health sector to highlight the outcomes of work undertaken under the HiAP approach to decision makers in the central agency of Government.

### Stages within the HIA and HiAP approaches

Both HIA and HiAP HLA involve a series of stages, and it is evident that elements of the HIA process were drawn upon by the SA Government when designing the HLA approach [46]. The strategies that are applied during each stage to achieve the goals of the work are, however, slightly different because of the different organisational positions of those implementing the work, and the tactical differences that this necessitates. Furthermore, the HLA approach continues to evolve in response to lessons learned during implementation [46]. The stages currently involved in both approaches are outlined in Table 3 and are aligned to show how they relate to each other. Table 3 makes it clear that much of the initial engagement undertaken during implementation of the HiAP approach occurs before the point at which a HIA approach is usually applied. More involved processes of screening and scoping are applied within the HIA approach. Many of the other aspects are similar, apart from the considerable bureaucratic navigation work that is required under the HiAP approach given its location within Government.

**Table 3:** Overview of stages used within the HIA and HiAP approaches

**New South Wales: Health Impact Assessment**	**South Australia: Health in All Policies**
	**1. Engage**
	• Develop relationship and discuss process, ensuring flexibility to cater to partners’ needs, with a focus on co-benefits.
	• Identify/clarify contextual issues.
	• Negotiate and agree on policy focus, taking political priorities into account.
**1. Screening**	• Identify resources.
• Identify elements of the proposal that could have an effect on health.	• Plan work and determine processes.
	• Establish evaluation criteria.
• Decide whether to pursue the process.	
**2. Scoping**	
• Decide on what, who, with, how and when the analysis will be performed.	
• Ensure focus is directed towards groups most at risk of being disadvantaged.	
**3. Identification/Analysis**	**2. Gather evidence**
• Review of the scientific literature.	• Undertake evidence gathering phase, using both qualitative and quantitative methods.
• Undertake consultation with experts and target population.	• Joint exploration and discussion.
• Make investigation and analysis.	• Reconcile perspectives.
	• Collaboratively shape conclusions and recommendations.
**4. Recommendations and reporting**	**3. Generate**
• Develop recommendations to reduce potential negative impacts and maximise positive effects on health, with a focus on improving health and equity as the drivers for these recommendations.	• Produce report and final recommendations, which are tailored to suit the relevant political and fiscal environments.
• Report on the process, findings and recommendations.	• Test ‘product’.
	**4. Navigate**
	• Navigate final report and recommendations through decision making processes and Government hierarchy, while emphasising co-benefits.
	• Provide briefings and presentations and organise necessary meetings.
	• Department of Health and partner agency Chief Executive to sign off.
	• Report to Cabinet task group Senior Officers Group.
**5. Evaluation**	**5. Evaluate**
• Review HIA process for improvement.	• Employ an external agency to evaluate project process, impact and outcomes and to identify process improvements.
• Evaluate actual policy or project impact if possible after 12 months where possible. In practice this is often difficult due to funding constraints.	
	• This is intended after all HLA projects, however, funding constraints mean that some projects may not be evaluated.

## Conclusion

This paper has demonstrated that the overall intent of HIA and HiAP is similar; that is, to provide evidence based recommendations to facilitate the development of policy and plans that will contribute positively to population health and equity. The techniques and tactics that are applied within each approach are, however, slightly different. Such differences arise from the institutional positions from which the approaches are implemented; with HiAP in SA applied from *inside* Government and HIA in NSW facilitated by an organisation who works in partnership with, but *outside* of, government and non-government organisations. HiAP operates as an engaged policy process that has the potential to influence all stages of the policy making cycle. The development of strong relationships between HiAP staff and staff from other Government departments is viewed as imperative to ensure the acceptability and institutionalisation of the HiAP approach and the ideas that arise from it. The acceptability of the HiAP approach within the SA Government is also bolstered by the mandate provided to it by the central Government agency. HIA operates without such support in NSW, and without being positioned as an integrated part of the policy process. As such, HIA is usually introduced later in the decision making process than HiAP. Relationship building occurs as a benefit or impact of the HIA process, although the main intent is to assess and predict the health and equity impacts of a proposal. This more technical intent of influencing a proposal and advocating for health and equity differs from the more tactical intent of the HiAP approach. Similar analyses could be undertaken to compare the findings presented in this paper with assessment of HIA in jurisdictions where it is implemented from within government rather than operationalised through external organisations.

While the close alignment of the HiAP approach with the current systems of the SA Government may increase the potential for influence, costs are also associated. In particular, the areas that are selected and the recommendations that are made are bounded by the priorities, agendas and political sensitivities of Government. This politicises the conduct of work under the HiAP approach as it is currently implemented in SA, and, in turn, limits the scope and breadth possible. It also puts constraints on who can collaborate to undertake the work, with little community input being possible. Due to its comparative distance from the NSW Government, the HIA approach is not bounded by political priorities in the same way. Those implementing HIA in NSW are able to work more freely with partners across all sectors of society (not just within Government) as well as generate recommendations that may not support, but rather challenge, the agenda of the current Government. The often explicit focus of HIA work on furthering population equity independent of political climate is an example of this. Conversely, within the HiAP approach the focus on equity is either made explicit or implicit depending on the political context surrounding particular pieces of work and depending on the broader Government agenda that governs the work. This does not mean that an equity agenda cannot be furthered through a HiAP approach; it can be, and indeed a focus on equity is evident in the broader foundations of a HiAP approach [47]. However, what is possible for HiAP in SA is highly dependent on the political choices and political agendas operating at a given time.

The analysis provided in this paper indicates that the differences that exist between HIA and HiAP approaches do not render them as incompatible or as, necessarily, in competition. Instead, their many points of connection highlight that both involve working towards similar goals by applying innovative ways of embedding health and equity concerns (implicitly or explicitly) within decision making systems. The diversity in the strategies that are applied within each approach provides a rich foundation for this kind of work in order to further efforts to achieve system level change which will optimise population health and its distribution.

## Competing interests

The authors declare that they have no competing interests.

## Authors’ contributions

TD led the development of this paper and was involved in all stages of the research, writing and manuscript preparation. PH contributed to the sections of the paper on HIA, assisted in examining the differences and similarities between HIA and HiAP, advised on the structure of the paper and undertook editing. CW and EH participated in brainstorming differences and similarities, contributed to writing the conference plenary that informs this paper and commented on drafts of the paper. FB, AL, DW and FH participated in brainstorming differences and similarities and commented on drafts of the paper. CM, DB and IK contributed to intellectual discussions that inform the paper and revised drafts of the manuscript. All authors read and approved the final manuscript.

## Pre-publication history

The pre-publication history for this paper can be accessed here:

http://www.biomedcentral.com/1471-2458/14/699/prepub
